# Urban forest biodiversity and cardiovascular disease: Potential health benefits from California’s street trees

**DOI:** 10.1371/journal.pone.0254973

**Published:** 2021-11-03

**Authors:** John J. Giacinto, G. Andrew Fricker, Matthew Ritter, Jenn Yost, Jacqueline Doremus

**Affiliations:** 1 Economics, California Polytechnic State University, San Luis Obispo, CA, United States of America; 2 Social Sciences, California Polytechnic State University, San Luis Obispo, CA, United States of America; 3 Biological Sciences, California Polytechnic State University, San Luis Obispo, CA, United States of America; Shenzhen University, CHINA

## Abstract

Enhanced immune functioning in response to biodiversity may explain potential health benefits from exposure to green space. Using unique data on urban forest biodiversity at the zip code level for California measured from 2014 to 2019 we test whether greater diversity of street trees is associated with reduced death from cardiovascular disease. We find that urban forests with greater biodiversity measured via the Shannon Index at the genus level are associated with a lower mortality rate for heart disease and stroke. Our estimates imply that increasing the Shannon Index by one standard deviation (0.64) is associated with a decrease in the mortality rate of 21.4 per 100,000 individuals for heart disease or 13% and 7.7 per 100,000 individuals for stroke or 16%. Our estimates remain robust across several sensitivity checks. A policy simulation for tree planting in Los Angeles based on our estimates suggests that if these relationships were causal, investment in planting for a more biodiverse set of street trees would be a cost-effective way to reduce mortality related to cardiovascular disease in urban areas.

## Introduction

Cardiovascular diseases, including heart disease, stroke, and hypertensive disease, are among the most preventable causes of death in the U.S. and worldwide [1–3] and cost the U.S. $199 billion in health care costs and $131 billion in lost job productivity annually [4]. Prescription drug treatment of cardiovascular disease has improved, shifting the policy focus to prevention [3]. Cost-effective ways to *prevent* cardiovascular may be situated outside traditional health care systems. Though they have existed in principle since at least the 19^th^ century, “green prescriptions,” or nature-based health interventions, are garnering attention and may help prevent cardiovascular disease [5]. For example, a short walk through a forest lowers blood pressure [6] and living in greener areas is associated with a reduced risk of cardiovascular diseases [7].

The mechanisms driving the association between biodiverse nature-exposure and health are unclear. Candidates include psychological restoration via the biophilia hypothesis [8] and dilution of the risk of infectious disease transmitted through an arthropod vector [9]. Summarizing the literature, Kuo [10] proposes a central pathway by which exposure to biodiverse nature would promote human health: *enhanced immune functioning*. Environmental biodiversity may contribute to enhanced immune function by improving the resiliency and diversity of human commensal microbiota [11–13]. If true, exposure to biodiversity could mitigate the autoimmune and inflammatory errors that contribute to cardiovascular diseases [13,14].

Despite potential links, to our knowledge no studies have assessed whether exposure to more biodiverse urban forests is associated with cardiovascular benefits. Most work has focused on green spaces, including absence and accessibility, or the size and number of trees in the landscape; the role of biodiversity is untested [15,16]. Within this literature, several studies find that exposure to trees is associated with better cardiovascular health. Donovan et al. [17] use changes in tree exposure from tree loss due to the Emerald Ash Borer and county-level mortality data and find a robust relationship for lower respiratory disease mortality and weaker results for heart disease mortality. A study with a similar design but individual-level data, which mitigates the risk of ecological bias, also found that tree loss from Emerald Ash Borer was associated with increased cardiovascular disease [18]. Using a cross-sectional design, several studies find that neighborhood greenness is associated with lower cardiovascular disease [19–21] and overall non-accidental mortality [22].

In our study, we assess the relationship between urban forest *biodiversity* and cardiovascular health, while controlling for total tree exposure, using California data from 2010–2018 at the zip code level. California’s urban forests are among the most diverse in the world. In concert with a diverse set of biomes and socioeconomic settings, California’s forests offer an unmatched setting for an initial assessment of the relationship between urban forest biodiversity and health outcomes. Our diversity measure, the Shannon Index, captures both the genus richness within a zip code and evenness of abundance across genus, e.g. the dominance of one or many genera. To calculate the Shannon Index, we use a unique dataset of individual street trees shared with us by a consortium of tree maintenance companies.

Our results show a statistically significant and robust negative association between genus diversity and cardiovascular disease mortality prevalence (heart disease and cerebrovascular disease). A one standard deviation increase in our diversity measure, the Shannon Index at the genus level, is associated with a decrease in the mortality rate of 21.4 per 100,000 individuals for heart disease or 13% and 7.7 per 100,000 individuals for stroke or 16%. We also investigate the potential causal drivers behind this relationship and fail to find support for the restoration or dilution hypotheses, leaving improved immune function as the most likely pathway.

Using our estimates, we simulate avoided mortality monetary benefits, net monetary benefits, and cost-effectiveness for a proposed tree planting quota for the Los Angeles, CA. With our simulation, we find that increasing the biodiversity of existing tree planting initiatives may yield a return of $2.70-$140.07 per dollar invested.

We join several studies that find that exposure to trees is associated with better cardiovascular health. Donovan et al. [17] use changes in tree exposure from tree loss due to the Emerald Ash Borer and county-level mortality data and find a robust relationship for lower respiratory disease mortality and weaker results for heart disease mortality. A study with a similar design but using individual-level data, which mitigates the risk of ecological bias, also found that tree loss from Emerald Ash Borer was associated with increased cardiovascular disease [18]. Using a cross-sectional design, several studies find that neighborhood greenness is associated with lower cardiovascular disease [19–21], and overall non-accidental mortality [22]. In our study, we assess the relationship between urban forest *biodiversity* and cardiovascular health, while controlling for total tree exposure. We find that the tree exposure itself is less important than the biodiversity of neighborhood trees, though the two are correlated. As the first paper, to our knowledge, to assess the relationship between urban biodiversity and cardiovascular health, we highlight this potential pathway by which greenness may affect health and offer a way to measure biodiversity, the Shannon Index.

## Conceptual framework

Aerts, Honnay, and Nieuwenhuyse [16] propose three hypotheses as to how biodiversity may affect cardiovascular disease: enhanced immune functioning, psychological restoration due to biophilia, and dilution of disease risk for vertebrates. In this section, we consider each in turn and then summarize our hypotheses.

Exposure to nature may affect health in many and varied ways, with *enhanced immune functioning* as a central pathway. Kuo [10] identifies 21 potential pathways and argues that among potential mechanisms, only enhanced immune functioning sufficiently (1) accounts for the empirically observed magnitude of nature’s impacts on health, (2) links to numerous specific observed health outcomes tied to nature, and (3) subsumes other identified pathways. Biodiversity may improve immune function through regulation of the composition of the human microbiome [16]. Immunological diseases such as asthma and diabetes, as well as ailments not traditionally considered immunological (obesity and depression) are gradually being found to be linked to the state of one’s microbiome [11,12,23–27]. Modification of the human microbiome may prevent chronic disease through enhanced immune response [28]. For example, among children, Hanski and colleagues [29] found that exposure to biodiversity was associated with greater diversity of commensal microbiota. Consistent with the “biodiversity hypothesis,” exposure to nature has been shown to reduce chronic autoimmune and inflammatory disorders and disease, including diabetes, cardiovascular diseases, and depression [29–31].

Experimental studies of exposure to nature offer indirect support of the link between biodiversity and improved immune function. Li [32] measured changes in substances vital to immune functioning, notably cancer-fighting natural killer (NK) white blood cells, before, during, and after sustained exposure to a forested area and found substantial increases in these beneficial compounds that lasted well after exposure. Park et al. [33] found that a short walk through a forest lowers blood pressure. These findings are reinforced by numerous other experimental studies of forest-bathing or “Shinrin-yoku” [34–36].

A second notable pathway by which urban greenspace may affect human health is through “psychological restoration” via stress reduction or recovery of directed attention [37,38]. Biodiversity has been shown to predict the restorative benefit of greenspace, suggesting diversity may play a role in the quality or extent of psychological restoration. Dubbed the “biophilia hypothesis,” in his 1984 book titled Biophilia, E.O. Wilson described restoration from biodiversity as “the innate tendency [in human beings] to focus on life and lifelike processes” [39]. Stemming from evolutionary history, humans may have innate preferences for the natural environment and exposure to biological diversity is restorative [40].

However, unlike the pathway of improved immune functioning, “psychological restoration” may also depend on an individual’s perception. The relation between biodiversity (actual and perceived) and indicators of psychological wellbeing is inconsistent [41], likely because biodiversity’s effect depends on an individual’s value judgments, as well as the time spent within the space, its location, and the activity performed within the space [42]. Health benefits from urban greenspace will depend on temporal exposure and spatial context. For example, for those spending most of the daylight hours away from their residential environment (i.e. work, commuting, etc.) the green characteristics of *that* landscape would be a more notable determinant of well-being [5].

Perhaps more subjective than any measure of psychological restoration would be a space’s stated aesthetic value, which plays a central role in the extent to which the space improves an individual’s subjective well-being [43]. Aesthetics, along with any subjective benefit derived by humans from biodiversity, does not follow a positive relationship with diversity measures. Aesthetic value is not as much a pure function of diversity as it is a product of one’s schema of what makes a green space attractive and enjoyable. Qiu et al. [44] find that the extent of one’s ecological knowledge serves as another moderator that shapes one’s perception of, preference for, and enjoyment of biodiversity.

The final mechanism by which biodiversity may affect human health is through the “dilution effect” hypothesis [16,45]. This hypothesis states that high vertebrate biodiversity reduces the risk of humans contracting diseases as the greater species mix dilutes the concentration of vertebrates often carrying harmful diseases transmittable to human-beings through an arthropod vector (i.e. Lyme Disease) [9]. However, evidence for the dilution effect is mixed when meta-analyses restrict to field studies [46].

Taken together, diversity within a stand of trees could enhance human immune functioning beyond that of a stand of a single genus through the commensal human microbiome. It could also improve restoration, reduce stress, and reduce exposure to arthropod-borne disease. However, it could also be insignificant in modifying the immune system, or detrimental to human health through some alternate pathway or reservoir [41]. Furthermore, since biodiversity is positively correlated with the number of trees in a forest, the separate effect of biodiversity from exposure to natural trees, as well as their interaction, is unclear. Our null hypothesis is that there is no significant relationship between biodiversity of urban trees, conditional on exposure to trees. The alternative hypothesis is that urban biodiversity is associated with human health outcomes.

## Materials & methods

### Study area

This study includes zip codes in the U.S. state of California from 2010–2018. California has 1,010 zip codes. Zip codes included in the analysis are the 857 zip codes with data available for each year for our Heart Disease sample and the 241 zip codes with data available for each year for our Stroke sample.

### Data sources

Our analysis uses data on health outcomes, urban forest characteristics, socio-demographic characteristics, and pollution characteristics. Table 1 presents summary statistics for the primary variables used within our analysis. For summary statistics on additional control variables used in our sensitivity analysis, see S1 Table in Supporting Information.

**Table 1:** Summary statistics.

	Mean	SD	Min	Max	N
Heart Disease	169.6	84.74	26.28	1,357	7,713
Stroke	49.30	28.54	0	657.7	7,713
Shannon Index	2.741	0.643	0	3.830	7,713
Tree per 100K Pop	0.158	0.178	6.57e-05	2.196	7,713

#### a) Health outcomes

We use California Department of Public Health’s Death Profile’s by zip code and year from 2010 to 2018. The data is a tabulation of the number of deaths to registered California residents, organized by mortality-type. Because the data are prepared for public use–aggregated, deidentified, and censored–informed consent was not required. We extracted deaths from Disease of the Heart (Heart Disease) and Cerebrovascular disease (Stroke). Place of death is unobserved: deaths of California residents that occurred outside the boundaries of the zip code of their legal address were still counted toward the total for their address zip code. Non-California residents who died inside of California are not reflected in zip code totals. The California Department of Public Health censors mortality counts below 11 deaths per zip code. Zip codes with small populations had many years of censored data. Like Khatama et al.’s [47] study of how Medicaid access affects cardiovascular mortality, we restrict our analysis to uncensored units. Among 1,010 potential zip codes, we restricted to the 857 zip codes that were never censored between 2010–2018 for Heart Disease and the 241 zip codes that were never censored between 2010–2018 for Stroke.

#### b) Urban forest characteristics

To calculate biodiversity, we use a unique dataset from several arborist companies who collectively make up the majority of arborist services in California. The shared tree inventory data was collected between 2014 and 2019 and includes one observation per tree sampled. The data come from a convenience sampling frame; the likelihood of sampling was not randomly assigned. The likelihood of sampling is the product of interactions between municipalities, private companies, individuals and the arborist companies servicing their trees. In terms of the sampling rate, we estimate it is around 3.6% based on data for Los Angeles: our data include about 25.5 thousand trees whereas the city of Los Angeles is estimated to have about 700 thousand trees (LA Bureau of Street Services 2020). Non-random sampling brings a risk of selection bias in the types of trees included in our sample. We address this concern with robustness checks that i) limit the sample to zip codes with many trees and ii) include socioeconomic controls. We also plot correlations between our biodiversity measure and household income and pollution in Supporting Information.

**Fig 1:**
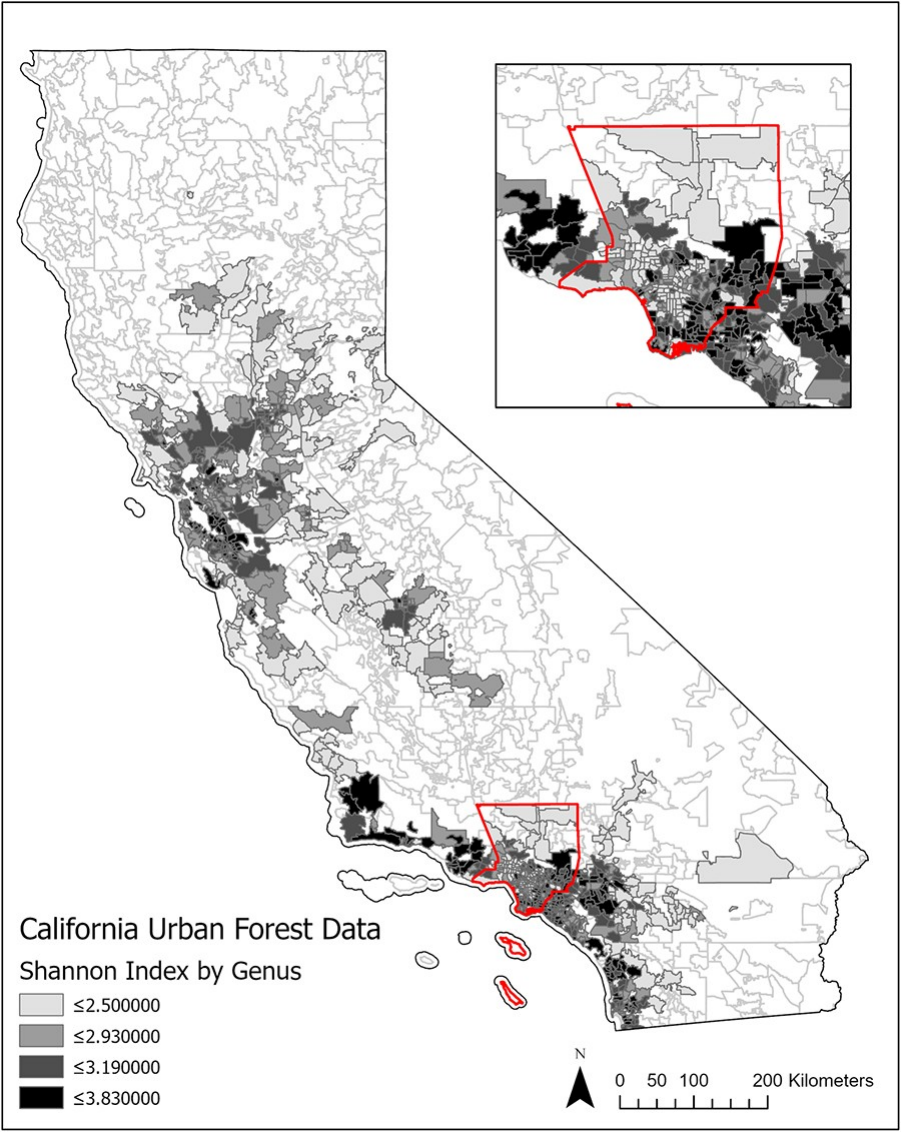
Variation in Urban tree diversity in California. Notes: Data for 857 zip codes in Heart Disease sample California from consortium of private tree maintenance companies.

Fig 1 shows the extent of our data in California by zip code tabulation area (ZCTA), a geospatial unit used by the US Census Bureau to facilitate relating census data to zip codes. We measure biodiversity using the Shannon Index, described in detail in the Measures section, for each ZCTA. We also control for exposure to trees using the number of trees sampled per 100,000 individuals by ZCTA. We merged these data with mortality rates calculated using the California Department of Public Health’s data.

Connecting health data with our urban forest characteristics comes with two empirical challenges. First, the urban forest biodiversity measure is based on sampling between 2014–2019, whereas the mortality data range from 2010–2018. This introduces measurement error that could bias our estimates downward. Second, the geographical boundaries of each dataset are not identical: the arborist data and population estimates are at the ZCTA level and mortality data is at the zip code level. Although U.S. Census-defined ZCTA boundaries are designed to correspond to US Postal Service zip-code boundaries, they often differ spatiotemporally as zip codes boundaries are moved, removed, or added without correspondence with ZCTA boundaries [48]. Some degree of mismatch joining across zip code and ZCTA is unavoidable but is assumed to be manageably small given that ZCTA is intended to be most compatible with zip code geographic boundaries. We begin our analysis in 2010 in order to maintain 2010 Census-defined ZCTA boundaries across observations. To facilitate exposition, we use the term zip code when referring to our geographic unit of analysis in the results section of the paper.

#### c) Sociodemographic and pollution characteristics

To calculate mortality and tree exposure rates per individual, we use two sources for zip code population. For 2010 we used population data from the US Census and for 2011–2016 we used the annual estimated population from the Five Year American Community Survey (ACS). For 2017 and 2018, where we lacked population data, we used the 2016 ACS population estimate. Neighborhood characteristics were taken from Opportunity Insight’s “Neighborhood Characteristics by Census Tract” which is an assembly of data sourced from the 2010 Census, 2012–2016 American Community Survey, 2000 Decennial Census, and Longitudinal Employer-Household Dynamics (LEHD). Characteristics were aggregated from census-tract to ZCTA. Given Census ZCTAs are designed to coincide with census-tract boundaries we assume there is no mismatch error in aggregating the data up to the ZCTA geographic level. Zip code level pollution data from 2018 were taken from CalEnviroScreen (CES) 3.0 and merged across ZCTA. The only variable used within our analysis is CES 3.0’s Pollution Burden Index, which is an average across percentiles of pollution indicators such as PM 2.5 concentration, drinking water contamination, traffic density, groundwater threats, and prevalence of hazardous waste facilities. When merging these data with the mortality rates and forest characteristics, we lose zip codes because of missing CES data and household income data from Opportunity Insights. The number of observations falls to 4,959 (551 zip codes) for heart disease and 1,449 (161 zip codes) for stroke.

### Measures

There are three key measures in our analysis: our outcome variable (mortality rate per 100,00 individuals), our control for exposure to trees (trees per 100,000 individuals), and our measure of biodiversity (Shannon-Weiner Index at the genus level).

Like Donovan et al. [17], we express mortality from cardiovascular disease as a rate as opposed to a count. We transformed raw mortality counts into rates of mortality per 100,000 individuals using ZCTA-level population data. To stay consistent, we also transform our exposure variable to trees per 100,000 individuals.

As discussed in the data section, mortality data correspond to the address of decedent, not the location of death. Thus, the relationship between exposure to biodiversity and mortality is approximated and based on the assumption that decedents spent some time near their address. Furthermore, people move. Our measure for biodiversity exposure likely reflects acute, recent exposure and may fail to reflect cumulative, chronic exposure. This limitation is common to other researchers who work with cardiovascular mortality rates [42,44].

Urban tree diversity is measured within each ZCTA at the genus-level using the Shannon-Weiner Index, Eq 1. In this expression, the share of trees within the first genus is $p_{1}=\frac{n_{1}}{N}$, N is the total number of trees in the ZCTA and *n*_1_ is the number of trees in the first genus of K total genera.


$$Shannon\;Index=-[p_{1}\;\ln\left(p_{1}\right)+p_{2}\;\ln\left(p_{2}\right)+\cdots +p_{K}\;\ln\left(p_{K}\right)]$$ (1)

The Shannon Index measures both evenness and richness across genera. Diversity was measured at the genus-level to capture greater phylogenic diversity as compared to measurement at the species-level. We assess the Shannon Index at the species-level in our sensitivity checks.

**Fig 2:**
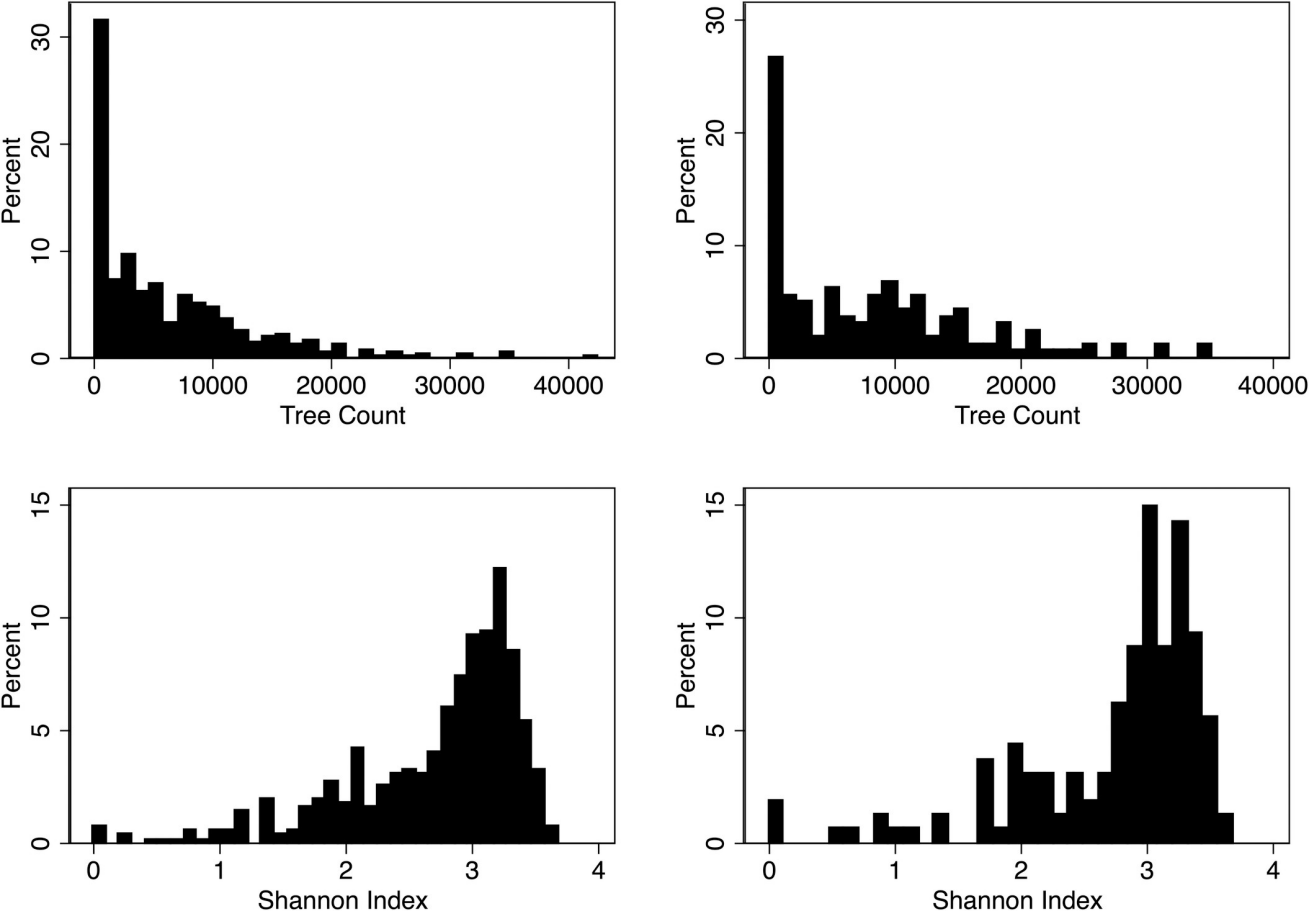
Histograms of Shannon index and trees per hundred thousand individuals. Notes: Data for 857 zip codes in Heart Disease sample California from consortium of private tree maintenance companies.

To account for the possibility that the tree characteristics do not have a linear relationship with heart disease and stroke mortality outcomes, we assigned binary variables for each quantile of the Shannon index. Thresholds are between 0 to 2.491 for the first quantile (N = 1,935), 2.495 to 2.930 for the second quantile (N = 1,926), 2.931 to 3.186 for the third quantile (N = 1,926), and 3.187 to 3.830 for the fourth quantile (N = 1,926). These cut offs are reflected in the heat map in Fig 1 and the distributions are shown in Fig 2.

### Analysis

The pathways through which biodiversity in urban forests contribute to human health appear numerous, nuanced, and likely intertwined in a complex web of other health determinants. Due to the complexity of the connections, causal chains are difficult to establish even for experimental studies like those of Li [32] and Thompson et al. [49]. Our approach is to assess macro-level (rather than micro) associations between urban biodiversity and human health outcomes. This approach carries risks: instead of identifying effects from the biodiversity of urban greenspace, there may be confounding unobserved factors correlated with biodiversity of urban greenspace or aggregation bias may lead to spurious correlations (for a humorous discussion of the risk of ecological fallacy within the context of biodiversity and health, see Salkeld and Antonlin [50].

To address this risk, we use a fixed effects panel OLS regression as our main specification, include several sensitivity checks, and make explicit that our analysis is descriptive and not causal. In our most parsimonious specification, we include fixed effects at the three-digit zip code level which controls for stable unobserved characteristics within the area of a 3-digit zip code level, *ϕ_z_*. This means that in our main specification, differences in mortality rates are identified off of variation in tree diversity and abundance within a three-digit zip code.


$$y_{it}=\alpha +\beta_{1}Shannon_{i}+\beta_{2}Trees_{i}+\Gamma X_{i}+\phi_{z}+\phi_{t}+\epsilon_{it}$$ (2)

We also include fixed effects by year, *ϕ_t_*, and, in sensitivity checks, sociodemographic controls *X_it_*. Standard errors are clustered by three-digit zip code to account for unobserved autocorrelation. However, spatial autocorrelation may be more complex and not correspond to three-digit code boundaries. Spatial noise could cause high t-statistics for the Shannon Index and trees per 100,000 individuals measures. To address this, we characterize the importance of spatial autocorrelation by calculating the Moran statistic and estimate the direct, indirect, and total effect of the Shannon Index on mortality, by year, using the *spreg* command in Stata 14 as a sensitivity check. The *spreg* command uses maximum likelihood to estimate a linear model that includes spatial lags of the Shannon Index and error term. We also use the *binscatter* command in Stata to create binned scatter plots [51] All calculations were done in Stata version 14.

To accommodate the possibility of non-linear relationships between biodiversity and mortality, we use an alternative specification that includes binary variables for each quantile of the Shannon index. The interpretation of the coefficients for these quantiles would be the difference in mortality when moving from the lowest quantile, Shannon I, omitted, to the quantile associated with the coefficient.


$$y_{it}=\alpha +\gamma_{1}Shanno{nII}_{i}+\gamma_{2}Shanno{nIII}_{i}+\gamma_{3}Shanno{nIV}_{i}+\beta_{2}Trees_{i}+\phi_{z}+\phi_{t}+\epsilon_{it}$$ (3)

To isolate the effect of biodiversity from that of trees, we also estimate the interaction of biodiversity quantiles with trees per 100,000 individuals.

We carry out a range of sensitivity checks against our parsimonious specification to further address risks from confounders. In addition to the regression with spatially correlated errors, described above, we assess the sensitivity of our estimates to: i) our measure of diversity, by assessing at the species level; ii) source of identifying variation, by estimating without fixed effects at the three-digit zip code level; iii) selection in arborist sampling, by restricting to zip codes with at least 1,000 trees; iv) omitted variables bias, by including socioeconomic and pollution burden controls and v) aggregation bias, by estimating separate subsamples by two-digit zip code.

Finally, we also estimate our main specification using weighted least squares, weighting by zip code population. Weighting may be justified if sampling is endogenous or the degree of intracluster correlation is low and the variance in the number of observations per cluster (zip code) is high [51]. These conditions are unlikely in our case. We found a high degree of intracluster correlation by Shannon Index, with a Moran statistic of 0.153 (p-value: 0.00) and sampling of tree data by the consortium of private arborists is unlikely to be correlated with heart disease or stroke mortality. However, comparing weighted least squares and ordinary least squares can be a useful diagnostic for model misspecification [52,53].

## Results

Our results begin with a graphical assessment of how differences in urban forest biodiversity correlate with heart disease and stroke mortality rates. We follow with analytical estimates of linear and non-linear specifications using ordinary least squares. After presenting our main results, we compare the linear estimate across several different specifications to assess the sensitivity of our primary estimates.

**Fig 3:**
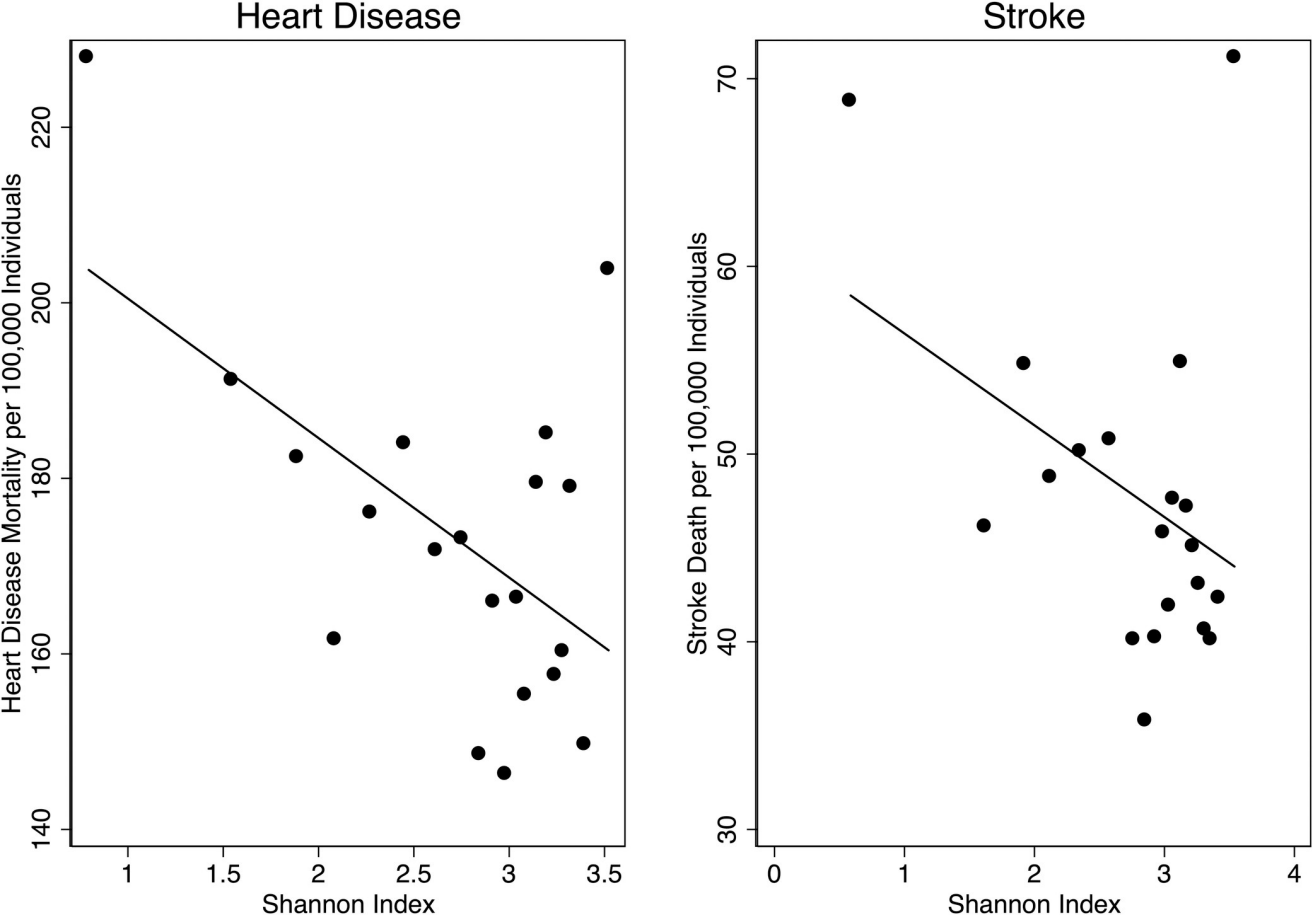
Urban forest diversity and mortality. Notes: Data for 857 California zip codes for Heart Disease and 241 zip codes for Stroke for 2010–2018 from California Department of Public Health. Shannon index calculated from data from consortium of private tree maintenance companies.

What is the shape of the raw relationship between urban forest diversity and mortality for heart disease and stroke? Fig 3 shows binned scatter plots of mortality rate per 100,000 people against the zip code’s Shannon Index.

As expected from the shape of the histograms of the Shannon Index in Fig 2, the binned scatter points in Fig 3 are concentrated among higher Shannon indices. There are fewer observations with very low Shannon Index values. For both Heart Disease and Stroke, we see a negative linear relationship between the Shannon Index and the mortality rate per hundred thousand people.

Next, we more formally estimate the relationship between the Shannon Index and mortality rate with a rich set fixed effects in a panel regression estimated using OLS. Unlike in our scatter plots, our regression controls for shared unobserved characteristics at the three-digit zip code level as well as the overall tree abundance per hundred thousand people within the zip code. Our results, reported in columns one and four of Table 2, have a similar pattern to Fig 3: the coefficient on the Shannon index is negative and precisely estimated for both heart disease and stroke mortality rates. The coefficients are equal to a decrease in the mortality rate per hundred thousand people of -33.5 (se = 11.0) for heart disease and -12.4 (se = 4.95) for stroke for an increase in the Shannon index of one. These coefficients are about half those found in the raw binned scatterplots in Fig 2. The estimate is more precise for Heart Disease, which has fewer censored zip codes, but the fit is better for Stroke.

**Table 2:** Urban forest diversity and mortality.

	(1)	(2)	(3)	(4)	(5)	(6)
	Heart Disease	Heart Disease	Heart Disease (interacted)	Stroke	Stroke	Stroke (interacted)
Shannon Index	-33.54**			-12.04*		
	(11.04)			(4.945)		
Shannon II		-34.74*	-11.63		-14.62	-8.008
		(14.74)	(12.46)		(10.58)	(9.540)
Shannon III		-49.30**	-27.90*		-17.76	-19.68*
		(15.84)	(13.03)		(10.15)	(9.573)
Shannon IV		-54.55**	-88.24**		-21.61*	-35.65**
		(16.18)	(26.03)		(9.875)	(10.86)
Trees per 100K pop	148.0	147.7	256.3	49.26	48.15	2.601
	(83.33)	(82.73)	(239.2)	(29.97)	(29.84)	(17.62)
Constant	238.1***	180.8***	179.1***	75.88***	56.88***	60.92***
	(26.73)	(11.82)	(10.91)	(13.78)	(8.395)	(8.027)
Observations	7,713	7,713	7,713	2,169	2,169	2,169
R-squared	0.178	0.177	0.229	0.275	0.270	0.363

Notes: Linear regressions for mortality per 100,000 persons within 55 3-digit zip code tabulation areas for heart disease and 42 for stroke, 2010–2018. Columns one and four measure urban forest diversity using the calculated Shannon index of street tree genus diversity. Columns two and five measure urban forest diversity using quantiles for the Shannon index where the lowest quartile is the omitted category. Columns three and six interact quartiles with Trees per 100K (interactions not reported). The regression includes year and 3-digit zip-code fixed effects, standard errors are clustered by 3-digit zip-code and reported in parentheses

*** p<0.001

** p<0.01

* p<0.05.

These estimates imply that increasing the Shannon Index by one standard deviation (0.64) is associated with a decrease in the mortality rate of 21.4 per hundred thousand for heart disease or 13% and 7.7 per hundred thousand for stroke or 16%. The estimate of the coefficient on trees per hundred thousand is unexpectedly positive, however it is imprecisely measured. Changes in an area’s Shannon index in response to tree planting or death will vary based on the underlying composition of the urban forest, thus making a direct and generalizable interpretation of the Shannon Index’s coefficient difficult. To aid in interpretation, in the discussion section we use our coefficient to estimate potential benefits of a proposed tree planting project in Los Angeles.

Though the plots in Fig 3 have a linear shape, the density of observations of the Shannon Index is weighted toward higher values. In columns two and five, we estimate a non-linear specification where the omitted category is the lowest quantile of the Shannon index (Shannon I), zip codes with Index values below 2.495. The interpretation of the coefficient for each quantile is the difference in the mortality rate for zip codes within the selected quantile as compared to those in the omitted quantile. These coefficients are precisely estimated for heart disease, with a potential threshold effect for the zip codes above the median (Shannon III and IV). We see a similar potential threshold effect for stroke, however the coefficients are estimated more precisely as the Shannon index quantile increases. Results hold when we interact biodiversity and trees per 100,000 individuals (columns three and six), suggesting biodiversity has an independent effect on mortality. The coefficient on the number of trees per hundred thousand people increases in magnitude but remains imprecise.

### Sensitivity analysis

In this section we present a sensitivity analysis of our linear estimate. In Table 3 we repeat our main estimate from the linear model in column one and consider seven alternative specifications to assess whether our estimates are sensitive to empirical design choices, as well as two additional tables in Supporting Information.

**Table 3:** Sensitivity of estimates.

	(1)	(2)	(3)	(4)	(5)	(6)	(7)	(8)
	Main	Species	No zip3 FE	Trim 1K	Pop Weighted	Control I	Control II	SAR (2018)
Panel A: Heart Disease Mortality Rate								
Coeff	-33.54**	-30.69**	-37.17**	-11.41	-19.01*	-39.07*	-37.65*	-39.2***
SE	(11.04)	(9.905)	(11.11)	(22.06)	(9.422)	(15.07)	(14.47)	(4.644)
N	7,713	7,713	7,713	5,301	7,713	4,959	4,959	857
R2	0.178	0.182	0.084	0.120	0.147	0.242	0.244	-
Panel B: Stroke Mortality Rate								
Coeff	-12.04*	-10.51*	-10.42*	2.790	-8.488*	-13.96*	-12.07*	-13.1***
SE	(4.945)	(4.505)	(4.672)	(10.58)	(3.539)	(5.888)	(5.272)	(2.860)
N	2,169	2,169	2,169	1,620	2,169	1,449	1,449	241
R2	0.275	0.274	0.147	0.185	0.247	0.424	0.436	-

Notes: Variations of linear regressions for mortality rate at the zip code-year level, measured in deaths per 100,000 individuals. Values reported are the coefficient on the relationship between the selected diversity measure and mortality rate, the corresponding standard error, and the number of observations used within the regression. All regressions except column three include 3-digit zip-code fixed effects. All regressions except column eight cluster standard errors by 3-digit zip-code. All regressions include year fixed effects.

*** p<0.001

** p<0.01

* p<0.05.

*** p<0.001, ** p<0.01, * p<0.05.

We first assess the sensitivity of our results to our biodiversity measure and spatial source of identifying variation. Column two of Table 3 uses the Shannon index for biodiversity at the species, instead of genus, level. The effect is slightly smaller but similar to the main estimate. Column three removes fixed effects at the 3-digit zip code level and thus identifies the correlation off of variation across California. Removing the 3-digit zip code fixed effects fails to change the estimate substantially.

Next, we use a blunt approach to assessing selection bias in our sample. Column four restricts the sample to zip codes with at least one thousand trees sampled in our dataset from private arborists. As shown in S1 Fig, there is a positive correlation between the Shannon Index and trees per 100,000 individuals. By imposing this threshold, we likely lose variation in the Shannon Index, reducing power. The estimate is still negative for Heart Disease, but it is near zero for Stroke and, unsurprisingly, both estimates are imprecisely estimated. This suggests that the zip codes with few trees are important when estimating our effect. This could be because of a threshold effect. In the non-linear specification in columns two and five of Table 2, the coefficients for Shannon II, III, and IV are not very different from one another. By removing the zip codes with the fewest trees, it is akin to comparing across these quartiles. We also lose power, having likely limited variation in the Shannon Index. Alternatively, given that the arborist sampling frame is not randomly assigned, the change in the coefficient estimate could reflect omitted variable bias if arborists are less likely to collect tree inventory data in zip codes with high mortality rates.

Another explanation of the difference in the trimmed and full sample would be heterogeneous treatment effects. Comparing OLS and weighted least squares helps assess if this is likely. Column five weights the OLS regression estimate by population, giving greater weight to zip codes with larger populations. This decreases the coefficient estimates by about half, though they remain negative and precisely estimated. As noted, differences in weighted least squares and ordinary least squares estimates may indicate model misspecification [52,53]. In our case, the direction of the effect and the precision of the estimate is similar across the two specifications, suggesting that our estimates are not sensitive to assumptions in the basic OLS model. However, we find that magnitude of the effect differs, suggesting some heterogeneity in the effect of biodiversity on the mortality rate.

To address omitted variable bias in our sample, columns six and seven add socioeconomic and pollution burden controls. The controls in column six include population density, median household income, mean commute time, and the share of the population that were Black, Hispanic, or Asian in the 2010 census. Adding these controls increases the magnitude of the estimate and slightly decreases the precision of the heart disease estimate. Column seven adds the same controls as column six as well as the zip code’s Pollution Burden Index. Adding the Pollution Burden Index slightly reduces the magnitude of the coefficients from that of column six.

Finally, column eight and S2 Table addresses spatial autocorrelation and S3 Table addresses aggregation bias. Column eight reports the total effect from a linear spatial autoregression model using data for 2018. The model includes spatial lags on the Shannon Index and error term and the total effect is the linear combination of the direct effect on mortality in a given zip code and indirect effects on correlated zip codes. The coefficient is similar in magnitude and precision to our main specification. Results for each year are very similar and are reported in S2 Table in Supporting Information. We also subsampled our data by two-digit zip code and report estimates using our main specification in S3 Table. We lose power by restricting the sample, and the estimates are less precise, particularly for Stroke. For Heart Disease, all but one of coefficients is negative and the association seems stable across two-digit zip code areas.

## Discussion

In this section we first discuss our main results and compare our effect sizes to those in the literature for greenspace and other interventions to prevent cardiovascular disease. Next, we explore possible mechanisms that might link biodiversity and health. Finally, to facilitate interpreting our results, we simulate benefits from a tree planting project in the city of Los Angeles.

We tested our null hypothesis that there was no association between biodiversity and mortality from cardiovascular disease and found that urban forest diversity and tree abundance are associated with lower mortality rates for Heart Disease and Stroke, and the effect was larger, more stable, and more robust for Heart Disease. Our results were insensitive to varying elements of the empirical design. These results are consistent with studies that assess the relationship between neighborhood greenness and cardiovascular mortality [19–21] and all-cause mortality [27].

Unlike for biodiversity, we failed to find a strong relationship between exposure to trees per 100,000 individuals and mortality. When assessing changes in heart disease mortality in response to changes in greenspace exposure from Emerald Ash Borer infestations, Donovan et al. [17] found a robust relationship for lower respiratory disease mortality and weaker results for heart disease mortality. For heart disease, the effect was not statistically significant. We, too fail to find a relationship between the two, however our analysis differs in three important ways. First, we are assessing a cross-sectional association whereas Donovan et al. [17] assesses a dynamic relationship within a causal framework. Second, our tree exposure variable is based on the number of trees sampled by our arborists, which may vary with the total number of trees per zip code, introducing measurement error. Finally, we include a measure of biodiversity, which is often but not always a function of the size of a forest stand. Given this relationship, measures of greenspace that do not include biodiversity may pick up spillover effects from biodiversity.

### Possible mechanisms

In our conceptual framework, we discussed three hypotheses proposed by Aerts et al. [16] as to how biodiversity may affect cardiovascular disease: enhanced immune functioning, psychological restoration due to biophilia, and dilution of disease risk for vertebrates. Given that we find that biodiversity is associated with health benefits, we might expect humans to be more aware of benefits from psychological restoration as compared to those from enhanced immune functioning or dilution of disease risk. If so, we would expect that higher biodiversity would be correlated with household income, as higher income households would have the means to invest in more restorative greenspace. S2 Fig plots binned scatter points for the Shannon Index against median 2016 household income. We fail to find a positive correlation between income and biodiversity, suggesting that, in this case, psychological restoration may not be the main pathway by which biodiversity could affect cardiovascular disease. This figure and the second panel, which shows a positive relationship between the Shannon Index and pollution burden, also allay some concerns that selection bias in our arborist data could be driving our results. The Shannon Index appears uncorrelated with income, which explains why adding controls did not greatly affect the coefficient in column six of Table 3, and the Shannon Index appears positively correlated with pollution burden.

To get a sense as to whether our results could be driven by the dilution hypothesis, we compared the county-level rate of Lyme disease per 100,000 person-years to the Shannon Index for 22 California counties with biodiversity data. S3 Fig in Supporting Information, a plot of binned scatter points, fails to show a negative relationship between the Shannon Index and incidence of Lyme disease. Instead, there is unexpectedly a weakly positive relationship, offering new evidence for the debate on the dilution hypothesis [46,54,55].

Given these analyses, enhanced immune functioning seems the most likely candidate as the pathway through which biodiversity might affect cardiovascular disease. As already stated, cardiovascular diseases encompassing diseases of the heart and stroke are closely tied to immune function [56–58]. The nature of our data is better suited to address the effects of acute exposure to tree diversity, as we do not control for movement of individuals within our dataset across time. Although this is a common issue [see 17,47], it limits the weight of our discussion on the long-run effects of biodiversity exposure on chronic cases of cardiovascular disease.

### Simulated benefits from tree planting in the city of Los Angeles

Estimated environmental benefits from planting a street tree in Los Angeles range from $38-56/year in the city of Los Angeles [59,60]. These and other benefit estimates focus on ecosystem services provided by urban forests, such as air and water purification, stormwater management, carbon sequestration, habitat provision, soil erosion protection, and the reduction of the urban heat-island effect [61–68]. Benefits from planting trees tend not to account for urban forest diversity, though there is a recognition that biodiversity may increase ecosystem resilience from environmental stressors [69,70].

To get a sense of the relative importance of biodiversity benefits in urban tree-planting initiatives, we simulated five planting scenarios for the Mayor of Los Angeles’ commitment to plant ninety thousand trees between 2020–2021 [71]. Our analysis is necessarily speculative, with uncertainty on several margins, the two most important of which are that our study only documents associations and it is unclear whether these relationships are causal and there is a risk that our data on forest characteristics is not representative. We present our simulation with the goal of encouraging a discussion about the relative value of increasing biodiversity in tree planting in preventing cardiovascular disease using different realistic planting scenarios, but want to be clear about its limitations.

In this spirit, for each scenario, we calculated the change in the heart disease and mortality rate and implied dollar values of these benefits. The five scenarios we considered fell into two types, planting a single genus or planting multiple genera. For planting a single genus, we compared planting the most commonly encountered genus in our data for the city of Los Angeles (“Top”) with planting the fifth most commonly encountered genus (“Fifth”). For planting multiple genera, we considered splitting new plantings evenly across the top two, five, and ten genera. Next, we scaled the ninety thousand trees down by 3.6% to be consistent with our sample. The result was 3,278 trees total to be planted in our simulation. To be consistent with the city’s emphasis on planting in areas with less urban forest, we targeted zip codes below the median, in terms of the number of trees observed in our data. The result was a total of 61 targeted zip codes (this includes censored zip codes not included in the analysis). We split our 3278 trees evenly among the 61 targeted zip codes and rounded to 50 trees per zip code.

**Fig 4:**
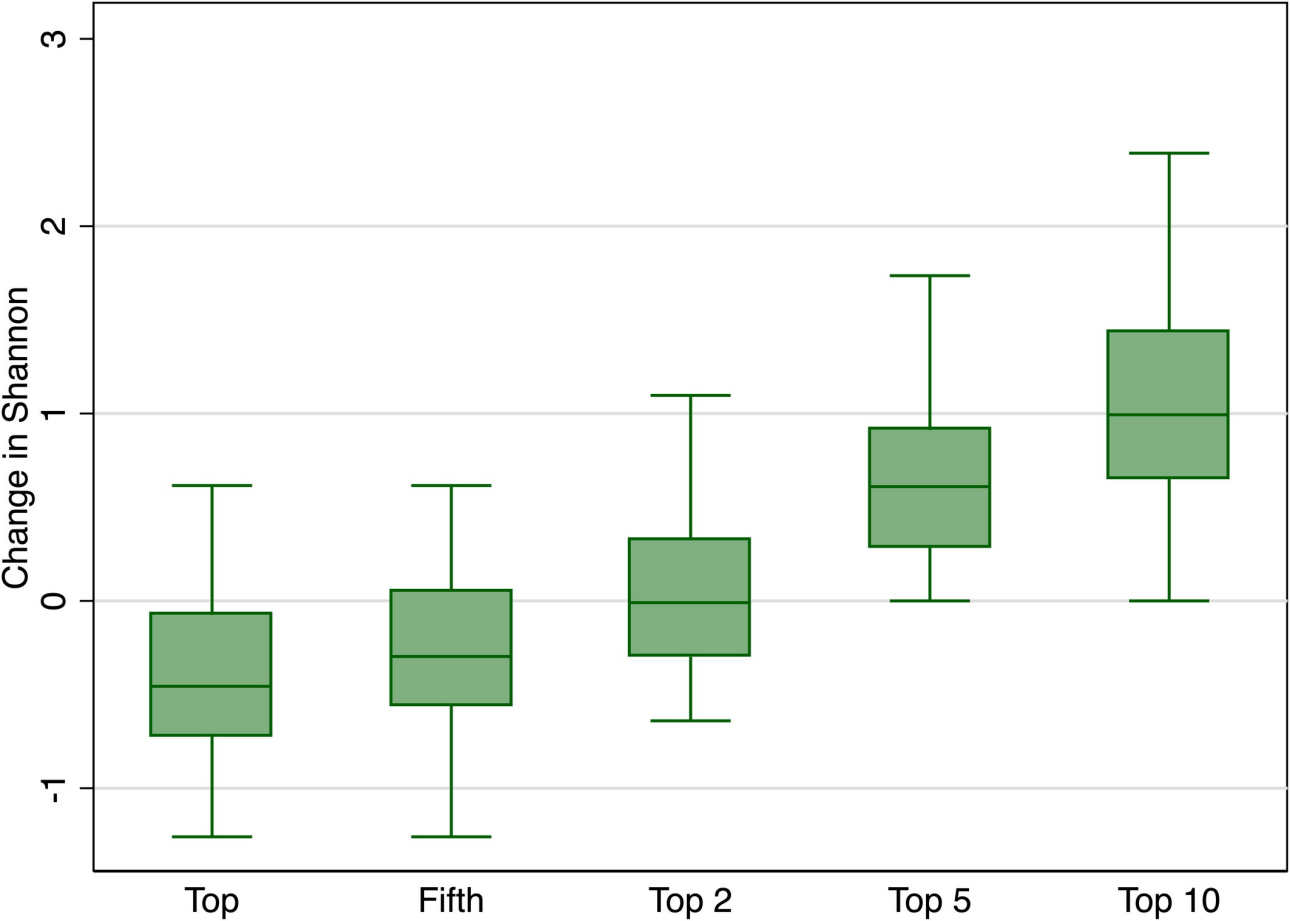
Change in Shannon index by number of genera targeted for simulated LA planting. Figure Notes: Data for 61 zip codes that are at least partly within the boundaries of the city of Los Angeles, California, and are in the bottom 50^th^ percentile in terms of total trees. The simulation evenly splits a total of 50 newly planted trees per zip code under five scenarios, each corresponding to a box plot: A single genus (the most common in LA, “top” and the fifth most common), as well as evenly splitting plantings across two, five, and ten genera. For example, in the top five genera scenario, each genus would increase the total trees by 50/5 = 10 trees. The Shannon Diversity Index is recalculated for each scenario and boxplots show the distribution in the change in the Shannon across 61 zip codes.

For each scenario, we calculated the Shannon index, shown in Fig 4. We used the change in the Shannon index and the estimated coefficient from the linear model (from Table 2) to calculate the implied change in the heart disease and stroke mortality rate and used the value of a statistical life of $5.6 million to get implied benefits [72]. Note that this is a simplification that likely overstates benefits. It would be more appropriate to use quality-adjusted life years. However, the dose-response function for exposure to biodiversity and prevention or delay of cardiovascular disease is unknown.

Keeping these important caveats and others in mind, results from our simulation for the five scenarios are reported in Table 4. We see that planting a single genus, whether the top or the fifth most popular, decreases evenness and exposure to biodiversity because the change in the Shannon Index is -0.39 or -0.30. The effect is similar for both scenarios, suggesting there are not large biodiversity benefits to uniform plantings of slightly less popular genus. In contrast, if plantings are spread evenly across just two genera, this increases exposure to biodiversity and brings potential avoided mortality benefits of $89 per zip code for heart disease and $32 per zip code for stroke. Further increasing the diversity of plantings to ten trees each of five genera results in very large benefits of $1,183 per zip code for heart disease and $425 per zip code for stroke.

**Table 4:** Avoided mortality benefits for Los Angeles tree planting initiative.

Scenario	Shannon	Heart Disease Rate	Heart Disease Benefits	Stroke Rate	Stroke Benefits	Total Benefits, Lo	Total Benefits, Hi
Top	-0.39	14	-$742	4.9	-$266	$892	$1,792
Fifth	-0.30	9	-$486	3.2	-$174	$1,240	$2,140
Top 2	0.054	-1.6	$89	-0.59	$32	$2,021	$2,921
Top 5	0.64	-22	$1,183	-7.9	$425	$3,508	$4,408
Top 10	1.1	-38	$2,041	-14	$733	$4,674	$5,574

Notes: Simulation of change in Shannon index and change in Heart Disease and Stroke Mortality Rates under five scenarios for 61 zip codes below the median number of trees per zip code in the city of Los Angeles. Simulation assumptions: Plant 50 trees per zip code and either target the most popular tree genus in the city of Los Angeles in our data or the fifth most popular, or split the fifty trees across the top two, five, or ten genera (so 25, 10, or five trees per genera). For each scenario, calculate the new Shannon Index, then the implied change in the Shannon Index from the no planting data. Use the coefficients from Table 2 for Heart Disease -33.54, and Stroke, -12.04, to estimate the change in the mortality rate for each planting scenario. Benefits are calculated by multiplying the change in the mortality rate by the value of a statistical life of $5.6 million.

### Net benefits from tree planting in Los Angeles

We add benefits from avoided mortality to benefits from eco-services from trees in Los Angeles, with the low estimate at $38 per tree per year to the high estimate of $56 per tree per year in the city of Los Angeles [59,60]. We can compare total annual benefits to total annual costs to calculate net benefits. Annualized lifetime planting and maintenance costs were estimated at $52–79 per tree per year [73,74], assuming a discount rate of 7%, allowing for extra costs associated with concrete tree wells, poor soil, and supplemental watering, and using an estimated Los Angeles street tree lifetime of 30 years based on allometric equations for eight species in our dataset. We begin by assuming there are no additional costs from increasing the diversity of street tree plantings by up to ten genera, though this may be unlikely if local nurseries fail to stock a sufficiently diverse set of seedlings. Given these assumptions, the total direct cost for planting fifty trees is $2,599–3,950 per zip code.

When considering total benefits, from avoided mortality and ecosystem services, increasing diversity increases the return on a tree-planting investment. For the lower planting cost estimate, net benefits (benefits–cost) are positive when planting is evenly split across two or more genera. For the higher cost estimate, net benefits are positive when planting is evenly split across five or more genera. Note that these benefits do not include those associated with self-reported positive emotions and restoration in physical and mental health [75,76], encouraged physical activity [77,78], lower obesity reports and improved social cohesion [6], and reduced asthma in young children [79] with exposure to greenspace or benefits to other species, such as birds [80].

### Cost-effectiveness of increasing biodiversity to combat cardiovascular disease

Next, we consider the cost-effectiveness of this intervention within the context of preventing cardiovascular disease. Cost-effectiveness compares the incremental change in the cost of a policy, as compared to the standard approach, and the incremental change in benefits. Given large uncertainties, assessing the cost-effectiveness of a policy to prevent cardiovascular disease is difficult to assess in the best of circumstances [81]. In our context, we are working outside of an experimental framework, so our estimates should be interpreted with caution.

We begin by assuming a tree-planting initiative without a biodiversity mandate is the standard approach. We simplify this to be planting the top genus, only. The incremental change in policy is increasing the biodiversity of plantings. The cost of increasing the diversity of plantings is unknown. A very rough estimate could be to include a 10–50% surcharge on the cost of a seedling to reflect administrative and transport costs to locate a vendor selling seedlings locally unavailable and transporting the seedlings to Los Angeles. This changes the planting cost range to $53–88 per tree or $2626–4380 per zip code, giving an incremental change of $27–417 per zip code. The gain would be the difference in benefits from planting only the top genus and the alternative scenario. This works out to $1,129–3,782 in total benefits from mortality from cardiovascular disease, where the lower number reflects the Top 2 scenario and the higher number the Top 10 scenario. In terms of cost-effectiveness, this is a return of $2.70-$140.07 for each dollar invested. The lower estimate is similar to results from a simulation that estimated medical cost savings in return for investing in parks and trails [82] and a comprehensive workplace wellness program [83]. This simulation suggests that if our effect sizes are replicated within a causal framework, increasing the diversity of tree planting may be a cost-effective way to prevent cardiovascular disease.

## Conclusion

Cardiovascular disease is the most common cause of mortality in the United States. Though management of blood pressure has improved over the last two decades, risk factors like obesity and diabetes remain challenging to address. Many population-based epidemiological studies have found an association between exposure to greenspace and lower cardiovascular risk. Numerous experimental studies of Shinrin-yoku and similar means of sustained forest exposure point to acute physiological changes after walking in a forest. Beyond the extent or *quantity* of general greenspace exposure, the *quality* of biodiversity may influence health outcomes via enhanced immune functioning and thus reduce mortality risks of diseases connected to proper immune functioning.

Our paper examines whether greater biodiversity within urban forests in California is associated with lower cerebrovascular and cardiovascular mortality. Using mortality data from 2010–2018, we find that increasing the diversity of an urban forest by one standard deviation (0.64) is associated with a decrease in the mortality rate of 21.4 per hundred thousand for heart disease or 13% and 7.7 per hundred thousand for stroke or 16%. The relationship is linear, though having very diverse forests may offer additional protection. We explore possible mechanisms and fail to find support that our effects are driven by psychological restoration or dilution of disease risk. We conclude that enhanced immune functioning seems the most likely pathway. As stated earlier, the role of actual biodiversity within green spaces and urban forests on human health outcomes remain highly unexplored. Our paper is the first to assess how biodiversity exposure is associated with cardiovascular health. Future research should use a causal framework to test the relationship between biodiversity and human health and potential mechanisms.

The strong association between urban forest diversity and reduced cardiovascular disease mortality suggests that tree-planting may be a useful policy lever to address cardiovascular disease. Benefits from planting street trees typically do not include avoided mortality from heart disease and stroke. A policy simulation of different planting scenarios for the city of Los Angeles found that even small efforts to increase planting diversity, such as splitting new plantings evenly across more than two genera, may be a cost-effective policy tool to reduce the risk of cerebrovascular and cardiovascular disease. Though our estimates include many uncertainties, we find that increasing the biodiversity of existing tree planting initiatives may beget a return of $2.70-$140.07 for each dollar invested. The lower estimate is similar in magnitude to the return on investment parks and trails and a comprehensive workplace wellness program.

Future research should use more comprehensive data on urban forest biodiversity, such as the future TreeKeeper data from LA, and an experimental design that allows for causal inference to directly test the relationship between biodiversity and prevention of cardiovascular disease and mortality. Work is also needed to better understand the dose-response function for chronic exposure to biodiversity when living near greenspace. Moving forward in this way could enrich our conception of “green prescriptions,” improve policy targeting to communities with inequitable access to biodiversity, and encourage cost-effective policy investments.

## Supporting information

S1 Fig Urban forest diversity and tree per hundred thousand people. Data for 857 California zip codes in Heart Disease sample from consortium of private tree maintenance companies.
(TIF)

S2 Fig Urban forest diversity, socioeconomic characteristics, and pollution burden. Data for 551 California zip codes in Heart Disease sample matched with income data from the American Community Survey and pollution burden data from the CalEnviroScreen 3.0. Shannon Index calculated from tree data from consortium of private tree maintenance companies.
(TIF)

S3 Fig Urban forest diversity and confirmed Lyme disease by CA county. Data for 22 California counties with forest biodiversity data. Lyme disease incidence calculated for period 2009–2018 by California Department of Public Health. Shannon Index calculated from tree data from consortium of private tree maintenance companies.
(TIF)

S1 Table Summary statistics for sociodemographics and pollution burden. Data for 551 California zip codes in Heart Disease sample matched with socioeconomic data from 2010 Census, 2012–2016 American Community Survey, 2000 Decennial Census, and Longitudinal Employer-Household Dynamics (LEHD), and CalEnviroScreen 3.0.
(DOCX)

S2 Table Spatial auto-regression model for each year. Estimates from a linear regression with spatially correlated error terms estimated using the *spregress* command (McMillan et al. 2008). Each column corresponds to a year of data. Coefficients reported are for the total effect of the Shannon index on mortality. Panel A is Heart Disease mortality and panel B is Stroke mortality. Estimates from 2018 also reported in main text. *** p<0.001, ** p<0.01, * p<0.05.
(DOCX)

S3 Table Subsample analysis by two-digit zip-code. Estimates from linear regression where the outcome variable is mortality rate per zip code-year, measured in deaths per 100,000 individuals. Column one repeats the main estimate. Columns two through seven are spatial subsamples for each two-digit zip code area, from 90 to 95. Regressions include year and 3-digit zip-code fixed effects, standard errors are clustered by 3-digit zip-code and reported in parentheses *** p<0.001, ** p<0.01, * p<0.05.
(DOCX)

S1 File (CSV)

S2 File (CSV)

S3 File (CSV)

S1 Data (DOCX)
