# Supplementary figures and images for: Urban forest biodiversity and cardiovascular disease: Potential health benefits from California’s street trees
Source: PLoS One. 2021 Nov 3;16(11):e0254973. doi: 10.1371/journal.pone.0254973 (PMC8565780; doi:10.1371/journal.pone.0254973)

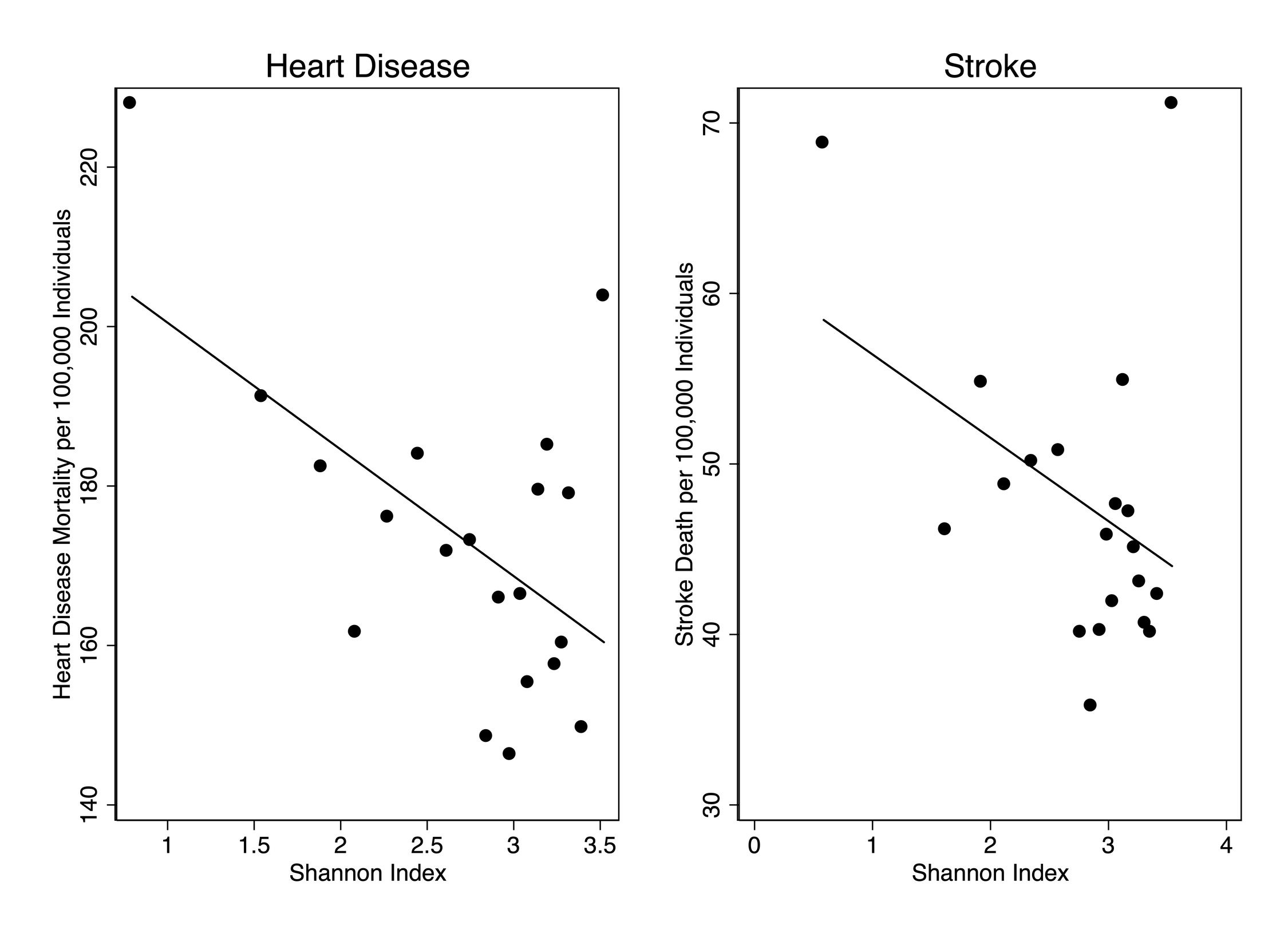

Supplement: S1 Fig — Data for 857 California zip codes in Heart Disease sample from consortium of private tree maintenance companies. (TIF) [file pone.0254973.s001.tif]

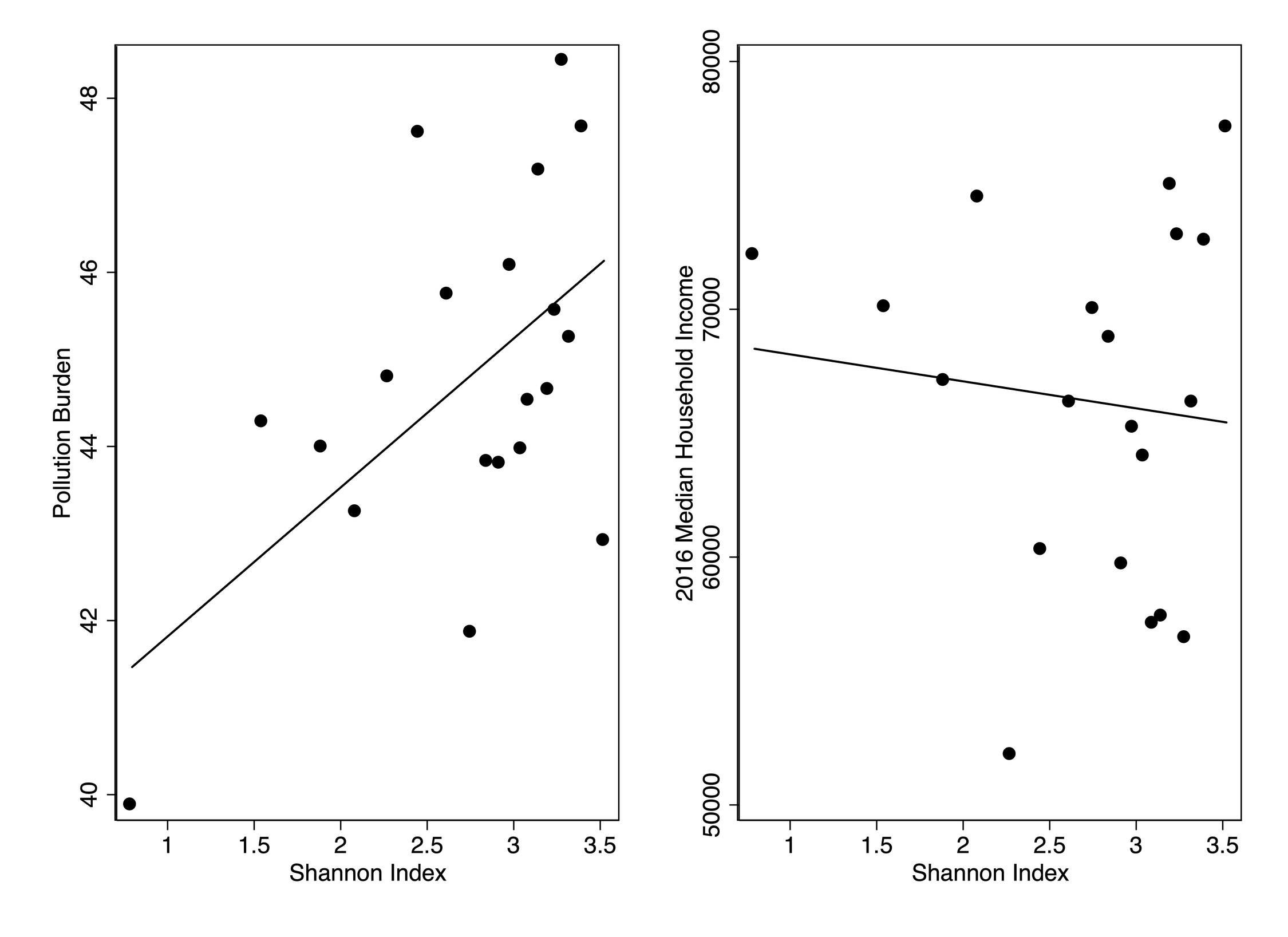

Supplement: S2 Fig — Data for 551 California zip codes in Heart Disease sample matched with income data from the American Community Survey and pollution burden data from the CalEnviroScreen 3.0. Shannon Index calculated from tree data from consortium of private tree maintenance companies. (TIF) [file pone.0254973.s002.tif]

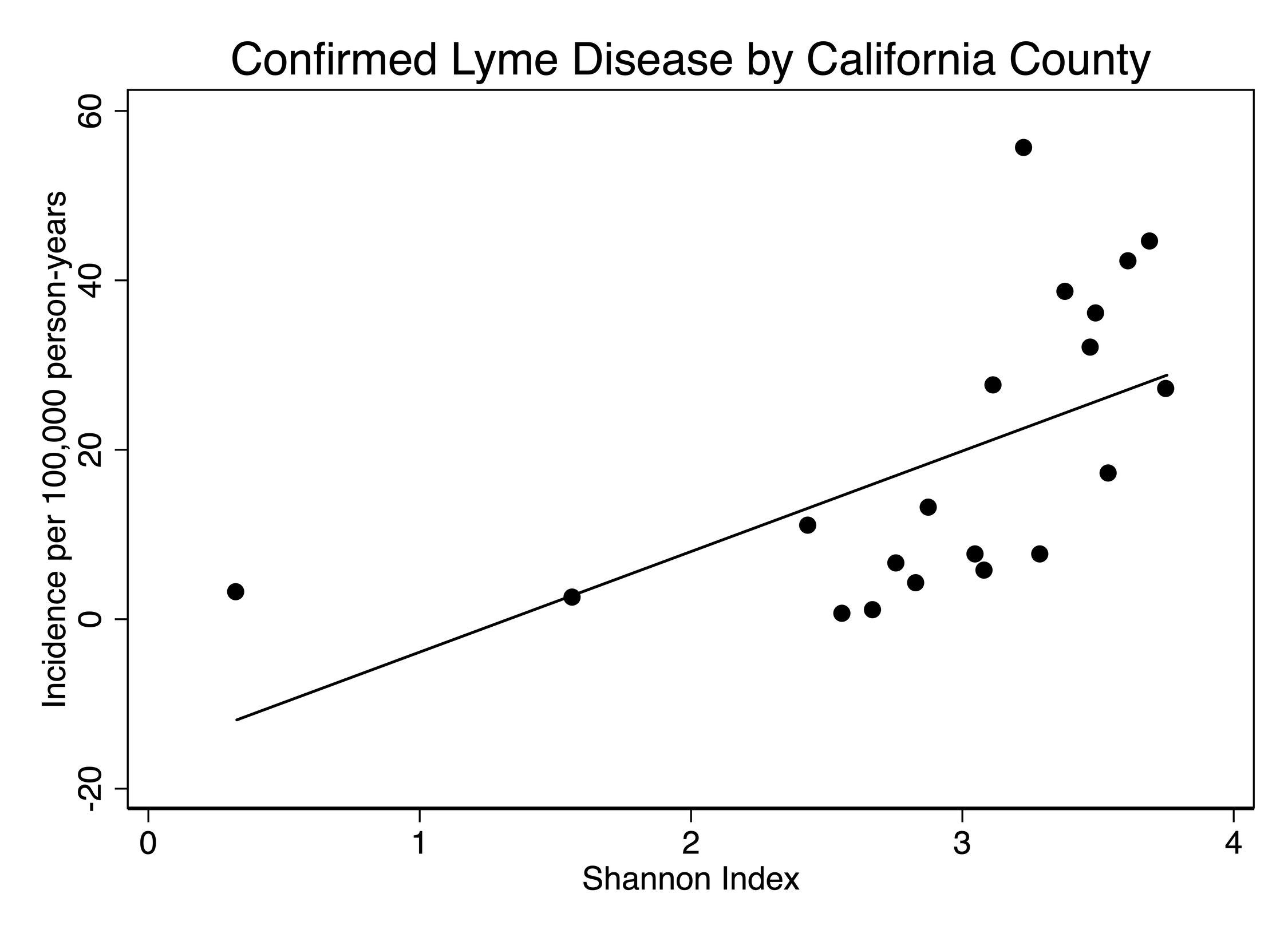

Supplement: S3 Fig — Data for 22 California counties with forest biodiversity data. Lyme disease incidence calculated for period 2009–2018 by California Department of Public Health. Shannon Index calculated from tree data from consortium of private tree maintenance companies. (TIF) [file pone.0254973.s003.tif]
