# Supplementary material for: Urban forest biodiversity and cardiovascular disease: Potential health benefits from California’s street trees
Source: PLoS One. 2021 Nov 3;16(11):e0254973. doi: 10.1371/journal.pone.0254973 (PMC8565780; doi:10.1371/journal.pone.0254973)
Supplement: S1 Table — Data for 551 California zip codes in Heart Disease sample matched with socioeconomic data from 2010 Census, 2012–2016 American Community Survey, 2000 Decennial Census, and Longitudinal Employer-Household Dynamics (LEHD), and CalEnviroScreen 3.0. (DOCX) [file pone.0254973.s004.docx]

**Table S1. Summary statistics for sociodemographics and pollution burden.**

|  | Mean | St.Dev | min | max | N |
| --- | --- | --- | --- | --- | --- |
| Median Household Income | 66309.3 | 26711.0 | 22741 | 198068 | 4,959 |
| Population Density | 8472.1 | 8277.0 | 2.165 | 79854.16 | 4,959 |
| Mean Commute Time | 29.4 | 4.61 | 16.65 | 47.23 | 4,959 |
| % Black Population | .06 | .078 | .002 | .586 | 4,959 |
| % Hispanic Population | .363 | .243 | .025 | .979 | 4,959 |
| % Asian Population | .101 | .116 | .002 | .681 | 4,959 |
| Pollution Burden Index | 44.8 | 10.91 | 11.07 | 75.37 | 4,959 |

Table Notes: Data for 551 California zip codes in Heart Disease sample matched with socioeconomic data from 2010 Census, 2012-2016 American Community Survey, 2000 Decennial Census, and Longitudinal Employer-Household Dynamics (LEHD), and CalEnviroScreen 3.0.
