# Supplementary material for: Urban forest biodiversity and cardiovascular disease: Potential health benefits from California’s street trees
Source: PLoS One. 2021 Nov 3;16(11):e0254973. doi: 10.1371/journal.pone.0254973 (PMC8565780; doi:10.1371/journal.pone.0254973)
Supplement: S2 Table — Estimates from a linear regression with spatially correlated error terms estimated using the spregress command (McMillan et al. 2008). Each column corresponds to a year of data. Coefficients reported are for the total effect of the Shannon index on mortality. Panel A is Heart Disease mortality and panel B is Stroke mortality. Estimates from 2018 also reported in main text. *** p<0.001, ** p<0.01, * p<0.05. (DOCX) [file pone.0254973.s005.docx]

**Table S2. Spatial auto-regression model for each year.**

|  | (1) | (2) | (3) | (4) | (5) | (6) | (7) | (8) | (9) |  |  |
| --- | --- | --- | --- | --- | --- | --- | --- | --- | --- | --- | --- |
|  | 2010 | 2011 | 2012 | 2013 | 2014 | 2015 | 2016 | 2017 | 2018 |  |  |
| Panel A: Heart Disease Mortality Rate | | | | | | | | | |  |  |
| Coeff | -37.52*** | -39.24*** | -35.5*** | -36.1*** | -35.5*** | -40.7*** | -41.8*** | -40.4*** | -39.2*** |  |  |
| SE | (5.160) | (5.440) | (4.809) | (4.78) | (4.600) | (4.993) | (4.680) | (4.648) | (4.644) |  |  |
|  |  |  |  |  |  |  |  |  |  |  |  |
| N | 857 | 857 | 857 | 857 | 857 | 857 | 857 | 857 | 857 |  |  |
| Panel B: Stroke Mortality Rate | | | | | | | | | |  |  |
| Coeff | -11.77** | -12.57*** | -8.688*** | -12.48*** | -9.75*** | -10.69*** | -9.70*** | -11.7*** | -13.1*** |  |  |
| SE | (3.022) | (2.648) | (2.451) | (2.73) | (2.632) | (2.776) | (2.670) | (3.137) | (2.86) |  |  |
| N | 241 | 241 | 241 | 241 | 241 | 241 | 241 | 241 | 241 |  |  |

Table Notes: Estimates from a linear regression with spatially correlated error terms estimated using the *spregress* command (McMillan et al. 2008). Each column corresponds to a year of data. Coefficients reported are for the total effect of the Shannon index on mortality. Panel A is Heart Disease mortality and panel B is Stroke mortality. Estimates from 2018 also reported in main text. *** p<0.001, ** p<0.01, * p<0.05
