# Supplementary material for: Urban forest biodiversity and cardiovascular disease: Potential health benefits from California’s street trees
Source: PLoS One. 2021 Nov 3;16(11):e0254973. doi: 10.1371/journal.pone.0254973 (PMC8565780; doi:10.1371/journal.pone.0254973)
Supplement: S3 Table — Estimates from linear regression where the outcome variable is mortality rate per zip code-year, measured in deaths per 100,000 individuals. Column one repeats the main estimate. Columns two through seven are spatial subsamples for each two-digit zip code area, from 90 to 95. Regressions include year and 3-digit zip-code fixed effects, standard errors are clustered by 3-digit zip-code and reported in parentheses *** p<0.001, ** p<0.01, * p<0.05. (DOCX) [file pone.0254973.s006.docx]

**Table S3. Subsample analysis by two-digit zip-code.**

|  | (1) | (2) | (3) | (4) | (5) | (6) | (7) |
| --- | --- | --- | --- | --- | --- | --- | --- |
|  | Main | 90 | 91 | 92 | 93 | 94 | 95 |
| Panel A: Heart Disease Mortality per 100,000 individuals | | | | | | | |
| Shannon | -33.54** | -6.416 | 9.107 | -81.29* | -37.49*** | -69.20** | -11.58 |
|  | (11.04) | (9.025) | (8.240) | (26.82) | (5.639) | (17.43) | (12.17) |
| Constant | 238.1*** | 168.5*** | 146.2*** | 345.8** | 249.2*** | 347.6*** | 208.3*** |
|  | (26.73) | (19.91) | (25.70) | (72.47) | (14.34) | (51.44) | (32.32) |
|  |  |  |  |  |  |  |  |
| Observations | 7,713 | 1,296 | 1,179 | 1,746 | 783 | 1,269 | 1,422 |
| R-squared | 0.178 | 0.132 | 0.133 | 0.363 | 0.199 | 0.100 | 0.198 |
| Panel B: Stroke Mortality per 100,000 individuals | | | | | | | |
| Shannon | -12.04* | 1.959 | 9.526 | -20.59 | -11.65* | -49.16 | 10.35 |
|  | (4.945) | (2.489) | (4.911) | (9.823) | (3.274) | (25.27) | (7.226) |
| Constant | 75.88*** | 31.35** | 8.466 | 91.66* | 74.76*** | 204.7* | 26.08 |
|  | (13.78) | (7.220) | (14.27) | (27.33) | (8.781) | (76.10) | (19.96) |
|  |  |  |  |  |  |  |  |
| Observations | 2,169 | 324 | 207 | 648 | 279 | 351 | 360 |
| R-squared | 0.275 | 0.206 | 0.262 | 0.496 | 0.361 | 0.206 | 0.268 |

Notes: Estimates from linear regression where the outcome variable is mortality rate per zip code-year, measured in deaths per 100,000 individuals. Column one repeats the main estimate. Columns two through seven are spatial subsamples for each two-digit zip code area, from 90 to 95. Regressions include year and 3-digit zip-code fixed effects, standard errors are clustered by 3-digit zip-code and reported in parentheses *** p<0.001, ** p<0.01, * p<0.05
