# Supplementary material for: Urban forest biodiversity and cardiovascular disease: Potential health benefits from California’s street trees
Source: PLoS One. 2021 Nov 3;16(11):e0254973. doi: 10.1371/journal.pone.0254973 (PMC8565780; doi:10.1371/journal.pone.0254973)
Supplement: S1 Data — (DOCX) [file pone.0254973.s010.docx]

*These ones don’t really have interpretative value, more so for use by ArcGIS and what not.*

objectid

zcta_key

latitude

longitude

shape_area

shape_len

*The following are more important for your purposes.*

**zipc** – The given zip-code of the given data point.

**zipc_3** – The first three digits of the zip-code of the given data point.

**year** – Year, corresponding to variables within the data that vary across time by year.

**population** – Population within the zip-code. For 2010, the data comes from the US Census. For 2011 – 2016 the data comes from the Five year American Community Survey (ACS). 2017 and 2018, lacking data, uses data we have for 2016.

*The following four are taken from the California Dept. of Public Health’s Death Profiles*

**htd –** Number of heart-disease related deaths of registered California residents of the given zip code from 2010 to 2018.

**can** – Number of cancer related deaths of registered California residents of the given zip code from 2010 to 2018.

**cld –** Number of chronic lower respiratory disease related deaths of registered California residents of the given zip code from 2010 to 2018.

**stk –** Number of stroke related deaths of registered California residents of the given zip code from 2010 to 2018.

**num_obs** – Count of individual tree observations within the given zip code.

**num_spec –** Count of species found within the given zip code.

**hhi_genus –** The Herfindahl-Hirschman Index at the genus level for a given zip code.

**hhi_spec -** The Herfindahl-Hirschman Index at the species level for a given zip code.

**shannon_spec –** The Shannon Index at the species level for a given zip code.

**shannon_genus –** The Shannon Index at the genus level for a given zip code.

*The following three are calculated as* ***`var’x = (`var’/population)*100000***

**htdx –** Heart disease mortality rate per 100,000 individuals.

**canx** – Cancer mortality rate per 100,000 individuals.

**stkx** – Stroke mortality rate per 100,000 individuals.

*The following four are for managing censored zip codes.*

**htdmin** – Minimum heart disease mortality count for a given zip code.

**htdcut** – Used to manage censored zip codes, any zip code containing an occurrence of a heart disease mortality count of 11 could then be dropped entirely.

**stkmin –** Minimum stroke mortality count for a given zip code.

**stkcut –** Same as htdcut but for stroke.
